# Decreased Hippocampal Neuroplasticity and Behavioral Impairment in an Animal Model of Inhalant Abuse

**DOI:** 10.3389/fnins.2018.00035

**Published:** 2018-02-06

**Authors:** Hanaa Malloul, Mohammed Bennis, Sara Bonzano, Giovanna Gambarotta, Isabelle Perroteau, Silvia De Marchis, Saadia Ba-M'hamed

**Affiliations:** ^1^Laboratory of Pharmacology, Neurobiology and Behavior (URAC-37), Faculty of Sciences Semlalia, Cadi Ayyad University, Marrakech, Morocco; ^2^Department of Life Sciences and Systems Biology, University of Turin, Turin, Italy; ^3^Neuroscience Institute Cavalieri Ottolenghi, Orbassano, Italy; ^4^Department of Clinical and Biological Sciences, University of Turin, Orbassano, Italy

**Keywords:** thinner inhalation, depression, anxiety, cognitive impairment, DG neurogenesis, BDNF

## Abstract

Thinners are highly toxic chemicals widely employed as organic solvents in industrial and domestic use. They have psychoactive properties when inhaled, and their chronic abuse as inhalants is associated with severe long-term health effects, including brain damage and cognitive-behavioral alterations. Yet, the sites and mechanisms of action of these compounds on the brain are far from being fully understood. Here, we investigated the consequences of paint thinner inhalation in adult male mice. Depression-like behaviors and an anxiolytic effect were found following repeated exposure in chronic treatments lasting 12 weeks. Both subchronic (6 weeks) and chronic treatments impaired learning and memory functions, while no changes were observed after acute treatment. To investigate possible molecular/structural alterations underlying such behavioral changes, we focused on the hippocampus. Notably, prolonged, but not acute thinner inhalation strongly affected adult neurogenesis in the dentate gyrus (DG), reducing progenitor cell proliferation after chronic treatments and impairing the survival of newborn neurons following both chronic and subchronic treatments. Furthermore, a down-regulation in the expression of BDNF and NMDA receptor subunits as well as a reduction in CREB expression/phosphorylation were found in the hippocampi of chronically treated mice. Our findings demonstrate for the first time significant structural and molecular changes in the adult hippocampus after prolonged paint thinner inhalation, indicating reduced hippocampal neuroplasticity and strongly supporting its implication in the behavioral dysfunctions associated to inhalant abuse.

## Introduction

Inhalant use disorder is defined as a pattern of inhaling hydrocarbon-based fumes, such as those found in solvents or paint thinner, for the purpose of altering the mental state (5th ed., DSM-5, American Psychiatric Association, 2013). Paint thinners are chemical mixtures of various halogenated and aromatic hydrocarbons (e.g., toluene, xylene, N-hexane and benzene), among the most common abused inhalants for their psychoactive and rewarding properties (Beckley and Woodward, 2013). Following thinner inhalation, hydrocarbon ingredients rapidly diffuse into the blood, cross readily through the blood-brain barrier thanks to their lipophilic properties, and produce a wide spectrum of adverse outcomes, including acute and chronic behavioral effects similar to those of other psychoactive drugs (Balster, 1998; Howard et al., 2011).

Significant cognitive impairments, including altered learning, memory, and attention, paralleled by a loss in white matter volume, particularly in the frontal temporal lobes, are among the symptoms reported following organic solvents chronic exposure in humans (Fornazzari et al., 1983; Rosenberg et al., 1988; Wang and Chen, 1993; Filley et al., 2004). Moreover, comorbidity with other mental impairments such as mood disorders, like major depression and antisocial personality disorder, has been reported in association with inhalant abuse (Sakai et al., 2004; Zubaran et al., 2010). Although inhalants are among the most common forms of abused substances worldwide, they remain the least studied of major drugs (Howard et al., 2011; Beckley and Woodward, 2013), and relatively little is known on their mechanisms of action. Over the last years, research on animal models has began to unravel selective effects of inhalants on common molecular targets (including several receptor systems such as NMDA and GABA; Bowen et al., 2006; Win-Shwe and Fujimori, 2010) and on specific neural circuits that underlie complex behaviors, including those involved in reward and cognition (e.g., mesolimbic and neocortical pathways; Permit et al., 2012; Beckley and Woodward, 2013; Fifel et al., 2014; Malloul et al., 2017). Nevertheless, further studies are needed to get deeper insights into the cellular/molecular mechanisms underpinning cognitive-behavioral deficits due to inhalant abuse.

In this study, we first assessed behavioral depression-related responses, anxiety, learning, and memory functions in adult mice following acute or prolonged thinner inhalation. Next, given the critical role played by adult hippocampal neurogenesis in learning and memory (Aimone et al., 2014) and its possible implication in disease conditions associated with cognitive impairment, depression, anxiety, and drug abuse (Kang et al., 2016), we characterized its alteration in paint thinner treated mice. Finally, we measured paint thinner treatment-induced changes in the hippocampus at the molecular level, analyzing the expression of the plasticity-related genes BDNF, NMDA receptor subunits and of the cAMP response element-binding protein (CREB). Our results show drastic hippocampal molecular/structural changes that are likely to be directly involved in the behavioral dysfunctions associated to inhalant abuse.

## Materials and methods

### Animals

Experiments were performed on 8-week-old male Swiss mice bred in the central animal care facilities of the Cadi Ayyad University, Marrakech (Morocco). Animals were maintained 5 per cage on a 12:12 light/dark cycle, with standard diet and water *ad libitum*. All animal procedures were in accordance with the European Communities Council Directive of November 24, 1986 (86/609/EEC), Recommendation 18/06/2007, Dir. 2010/63/UE. The study received also the approval of the Council Committee of research laboratories of the Faculty of Sciences, Cadi Ayyad University of Marrakech.

### Paint thinner exposure

We exposed mice to paint thinner (Sodas, Mohammed, Morocco), whose chemical composition, determined in our previous study (Fifel), includes more than 25 distinct molecules among which the most representative are Toluene (24.46%), Xylene (15.47%), Benzene (10.67%), Dichloromethylene (6.34 %), and Acetone (5.55%).

Experimental paradigms used comprise acute (1 day), subchronic (6 weeks), and chronic (12 weeks) treatments. For each experimental paradigm mice were randomly divided into 3 groups (two treated and one control; *n* = 5 each) and placed in a whole-body inhalation chamber as previously described (Fifel). Briefly, 200 or 400 μl of liquid thinner was added to a filter paper located on a glass petri dish covered by a wire mesh on the inhalation chamber floor to obtain an estimated thinner concentration of 300 ppm or 600 ppm respectively. Mice were put in the whole inhalation chamber twice a day (first exposure between 8:00 and 9:00 a.m. and second exposure 8 h later) for two sessions of 15 min (thinner was renewed at the beginning of each session), separated by 5 min interval in which mice were returned to the home cage. The control group was maintained in the exposure chamber for the same periods and conditions as for treated mice, but without thinner.

### Behavioral tests

To assess depressive-like behaviors we used the tail suspension test (TST; Steru et al., 1985) and forced-swim test (FST; Porsolt et al., 1977); anxiety-like state of mice was evaluated by means of the open field test (OFT; Sahay et al., 2011) and the elevated plus maze test (EPMT; Torres and Escarabajal, 2002); memory retention was assessed by the step-through passive avoidance task (SPAT; Ogren and Stiedl, 2013), and recognition memory by the object recognition test (ORT; Antunes and Biala, 2012). The OFT, EPMT, and ORT were recorded and analyzed using Ethovision XT Noldus 8.5 video tracking program (Noldus, The Netherlands) connected to a video camera (JVC). The behaviors in TST, FST, and SPAT were video-recorded (Samsung SCO-2080R) and measured manually using the event-recording function in the video-tracking software (Debut video capture software, NHC). All the tests were performed between 8:00 and 12:00 a.m. to avoid any circadian related fluctuation in the performance of the animals, starting from the day after the last thinner exposure. To reduce the number of animals used, both in the subchronic and chronic treatments the same mice were tested for either FST, OFT, and ORT or for EPMT, TST, and SPAT with an interval of 1 day between each assay.

#### Tail suspension test

Mice were suspended from a plastic rod mounted 50 cm above the surface by fastening the tail with adhesive tape. Immobility, defined as the absence of any limb or body movements, except those caused by respiration, was measured during 6 min.

#### Forced swim test

The mice were gently placed in a clear rectangular glass container (20 × 50 × 30 cm) filled with water at 30°C. Climbing (i.e., front paw movements against the tank wall bringing part of the body out of the water) and immobility (i.e., no movements of limb, tail, or head) of the mice were scored over a period of 6 min. Immediately after, each animal was removed from the water, towel-dried, and returned to its home cage. The water was changed and the container was cleaned in between each experimental animal.

#### Open field test

The apparatus used for this test consisted of a simple square enclosure field (50 × 50 × 50 cm). A 75 W lamp was placed in porthole diffusing light and located at 200 cm from the device allowing the center of the apparatus to be under a dim light (100 lx). At the beginning of each session, mice were placed in the central part of the arena and the total time spent into center and border was determined over a 10 min period. The center zone is 17.5 cm from the wall of the maze, corresponding to the standard area (Malloul et al., 2017). The apparatus was cleaned with a 75% ethanol solution in between each trial to remove any trace of odor.

#### Elevated plus maze test

The maze consisted of two acrylic open arms (50 × 5 cm) and two enclosed arms (50 × 5 × 15 cm) connected to a common central platform (5 × 5 cm). The maze floor and the side/end walls (15 cm height) of the enclosed arms were made of clear Plexiglas. The apparatus was set 50 cm above floor level and was under an approximate brightness of 200 lx. Each mouse was placed in the center facing an open arm and left to explore the maze for a single 5 min recorded session. The percentage of time spent in the open arms was analyzed by calculating the “time spent in the open arms” divided by the “total time spent in both the open and enclosed arms.” Maze was cleaned in between each trial.

#### Step-through passive avoidance task

The SPAT apparatus consisted of a bright (200 lx) and a dark equally sized Plexiglas compartments (30 × 25 × 25 cm), with independent electrical grid floor, and connected by a guillotine door (10 × 8.5 cm). The training session was carried out 24 h after the last exposure to thinner. In the training session, each mouse was placed in the light chamber, and as soon as the animal entered the dark chamber, the door was closed and an unavoidable electric foot shock (0.5 mA, 5 s duration) was delivered by a shocker through the grid floor. The animal was removed from the chamber 10 s after receiving the shock and replaced in its home cage. The retention of the avoidance response was tested 24 h later. In the test session, each mouse was placed into the light chamber and the time taken to enter the dark compartment was measured as step-through latency. The mice that did not enter the dark chamber during the cut-off time (180 s) were removed from the box and assigned a ceiling score of 180 s. Short latencies indicate poor retention.

#### Object recognition test

Mice were first habituated in the testing open field arena (50 × 50 × 50 cm) in the absence of any objects during 10 min, 24 h before starting the task. The objects to be discriminated were in plastic with three different shapes: cube, pyramid, and cylinder. They were 3.5 cm high and could not be displaced by the mice. During the training session, two objects selected randomly with different shapes were presented to each mouse for 10 min. The open field area and objects were cleaned with 75% ethanol between trials to prevent the buildup of olfactory cues. The 10 min-long test session was performed 24 h after training; one of the same objects and a novel one were presented to the trained mouse. The interaction of the mouse with each object, including approaches and sniffing, was recorded by the video tracking system. If the mouse had memory retention for an old object, it would show preference to the novel object during testing. The percentage preference was defined as the “time spent investigating a specific object” divided by the “total time spent investigating both objects.”

### 5-bromo-2-deoxyuridine injections

For assessing proliferation, acute, subchronic and chronic treated mice were intraperitoneally injected with a single pulse of 5-bromo-2-deoxyuridine solution (BrdU; 100 mg/kg in 0.1 M Tris, pH 7.4; Sigma-Aldrich) 24 h after the last thinner exposure and sacrificed 24 h later. For evaluating newborn cell survival in the DG, BrdU was injected for seven days (one injection/day; 50 mg/kg in 0.1 M Tris, pH 7.4; Sigma-Aldrich) starting from the beginning of the subchronic treatment and on week 6 for the chronic treatment. For both treatments, mice were allowed to survive for further 5 weeks following the last BrdU injection.

### Tissue preparation and sectioning

Mice were deeply anesthetized by an intraperitoneal injection of a sodium pentobarbital (>90 mg/kg) and perfused transcardially with 0.9% saline, followed by 4% paraformaldehyde in 0.1 M phosphate buffer (pH 7.4). Brains were removed from the skull, post-fixed overnight in the same fixative solution, cryoprotected in a 30% sucrose solution, frozen and cryostat sectioned (Leica Microsystems, Milan, Italy). Forty-micrometer thick free-floating coronal sections containing the DG were collected serially in multiwell dishes (12 wells/animal). Sections were stored at −20°C in antifreeze solution until use.

### Immunofluorescence

Sections were incubated 48 h at 4°C in primary antibodies diluted in 0.01 M phosphate-buffered saline (PBS, pH 7.4), 0.5% Triton X-100 and 2% normal serum of the same species as the secondary antibody and then incubated for 2 h in the appropriate secondary antibodies. The primary antibodies used were: anti-BrdU (1:3000, rat, AbD Serotec, code number OBT0030CX), anti-Ki-67 (1:1,000, rabbit, Novocastra, code number NCL-Ki67p), anti-Ki-67 (1:500, mouse, BD Pharmingen, code number 550609), anti-PH3 (1:1,000, rabbit, Millipore, code number 2066052,), anti-GFAP (1:2,000, rabbit, Dako, code number Z0334,), anti-DCX (1:1,500, goat, Santa Cruz Biotechnology, code number Sc-8066), anti-NeuroD1 (1:400, goat, Santa Cruz Biotechnology, code number Sc-1804), anti-NeuN (1:1,000, mouse, Chemicon, code number MAB377), anti-Caspase-3(1:300, rabbit, Cell Signaling Technology, code number D9661S). For BrdU immunostaining, sections were pre-treated with 2N HCl for 30 min at 37°C, and neutralized with borate buffer (pH 8.5) before incubation with anti-BrdU. For DCX, Ki67 and NeuroD1 reactions, sections were pre-treated with sodium citrate buffer (pH 6.0) for 10 min at 95°C. For immunofluorescence double/triple-staining, the sections were incubated in a mixture of primary antibodies (made in different species) and appropriate blocking sera. For multiple labeling with BrdU, sections were first incubated 48 h at 4°C in anti-NeuN and anti-GFAP primary antibodies and the appropriate serum, and then for 2 h at room temperature in secondary antibodies. Sections were then processed for BrdU detection following the protocol described above. Secondary antibodies were used as follows: donkey anti-rabbit and anti-rat Cy3-conjugated (1:800, Jackson ImmunoResearch, code number 712-165-153); donkey anti-goat and anti-mouse AlexaFluor 647-conjugated (1:600, Jackson ImmunoResearch, code number 705-605-147 and 705-605-151); donkey anti-mouse AlexaFluor 488-conjugated (1:400, Jackson ImmunoResearch, code number 715-545-151). For immunofluorescence reactions, sections were incubated in the appropriate secondary antibody/ies for 2 h at room temperature (RT), counterstained with the nuclear dye 4′, 6-diamidino-2-phenylindole (DAPI) (1:1,000) then mounted on gelatine-coated slides, air dried and cover-slipped with anti-fade mounting medium Mowiol (4-88 reagent, Calbiochem 475904).

### Microscopy and quantifications

All cell counts were conducted blind with regards to the experimental group. Cell counting and image analysis were performed on either a Nikon microscope coupled with a computer-assisted image analysis system (Neurolucida software, MicroBrightField) or a TCS SP5 confocal microscope (Leica). To estimate the volume of the DG granule cell layer (GCL), we used one series of sections out of 12 per animal (i.e., *n* = 5/6 sections representative of the entire DG). The boundaries of the GCL were drawn based on DAPI staining and the GCL area of each DG was automatically calculated by Neurolucida software. The total volume of the GCL was estimated by applying the Cavalieri method (Prakash et al., 1994). For each analysis, one series of sections (*n* = 5/6 sections) per animal was used and the density of antigen-positive cells was calculated using the formula D = N/[(A^*^t)/10^6^], where N is the number of counted cells, A is the layer area (μm^2^) t is the thickness of the section analyzed, and expressed as the number of positive cells per mm^3^. Confocal image z-stacks were captured through the entire slice thickness at 1 μm optical steps and used for double/triple-labeled cell counts. The mitotic index was calculated as the fraction of mitotic cells (i.e., PH3-positive) among the Ki-67^+^cell population in the SGZ/GCL. The fraction of newly generated cells (BrdU^+^ cells) co-expressing either GFAP or NeuN among all BrdU^+^ cells was calculated by using manual cell counting on ImageJ software on confocal images. Double BrdU/GFAP labeled cells were classified as radial-glia or astrocytes based on morphological criteria (Gebara et al., 2016). To assess the phenotype of apoptotic cells in the DG, double/triple-labeled cells for Caspase-3, DCX, and/or NeuN, were systematically analyzed throughout the rostral-caudal DG axis by examining two series of sections per animal. Confocal pictures and reslicing were assembled into panels using the Inkscape 0.91 software.

### RNA isolation, cDNA preparation, and quantitative real-time PCR

24 h following the final thinner inhalation of chronic treatment, RNA was extracted from hippocampus from independent groups of control and 600 ppm exposed mice (*n* = 5 each). Total RNA was isolated using RNeasy® Mini Kit (Qiagen) according to the manufacturer's instructions. Retrotranscription (RT) of 1 μg total RNA was carried out in a 25 μl reaction volume containing: 1x RT-Buffer, 0.1 μg/μl bovine serum albumin (BSA), 0.05% Triton, 1 mM dNTPs, 7.5 μM Random Hexamer Primers, 40 U RIBOlock, and 200 U RevertAid® Reverse Transcriptase (all RT ingredients were provided by Thermo Scientific). The reaction was performed 10 min at 25°C, 90 min at 42°C, 15 min at 70°C. Quantitative real-time PCR (qRT-PCR) was carried out using an ABI Prism 7300 (Applied Biosystems) detection system. cDNA was diluted 10 fold in nuclease-free water and 5 μl (corresponding to 15 ng starting RNA) were analyzed in a 20 μl reaction volume, containing 1 × iTaq Universal SYBR Green Supermix (BioRad) and 300 nM forward and reverse primers. Dissociation curves were routinely performed to check for the presence of a single peak corresponding to the required amplicon. Analyses were performed in technical duplicate and biological quintuplicate.

Data from qRT-PCR experiments were analyzed using the −ΔΔCt method for the relative quantification. The threshold cycle number (Ct) values were normalized to the geometric average of two endogenous housekeeping genes: TBP (TATA box Binding Protein) and UbC (UbiquitinC), as suggested by Vandesompele et al. (2002). As calibrator, the CT average of control samples was used. All normalized relative quantitative data are shown as 2^−ΔΔ*CT*^. Primers were designed using Annhyb software (http://www.bioinformatics.org/annhyb/), possibly on different exons separated by a large (>1000 bp) intron, and were synthesized by Invitrogen. Primer sequences are reported in Supplementary Table 1.

### Protein extraction and western blot analysis

After RNA extraction with RNeasy® Mini Kit (Qiagen) total proteins were precipitated with acetone from lysates according to the manufacturer's instructions. In the final passage, the protein pellet was re-suspended in 300 μl boiling Laemmli buffer (2.5% SDS, 0.125 M Tris-HCl, pH 6.8) by 1 min sonication with Bandelin Sonoplus GM2070/GM2200 (Bandelin Electronic, Berlin, Germany), denatured at 100°C for 3 min and spun at room temperature for 20 min at 12000 rpm to discard cell debris. Protein concentration was determined using the Bicinchoninic Acid (BCA) Protein Assay Kit (Sigma-Aldrich) on 1:4 diluted proteins to avoid detergent interference. Equal amounts of proteins (30 μg) were loaded into each lane. Proteins were resolved by 8% SDS-PAGE, transferred to a supported nitrocellulose membrane (Biorad, code number 162-0093) and blocked 1 h at 37°C in 1X TBST containing 5% nonfat milk. Primary antibodies used are anti-phospho-CREB Ser 133 (1:1000, rabbit, Cell Signaling, code number 9198), anti-total-CREB (1:1000, rabbit, Cell Signaling, code number 9197,) and anti-GAPDH (1:20000, mouse, Ambion, code number AM4300). Secondary antibodies used are horseradish peroxidase linked anti-rabbit (1:20000, GE Health, code number NA934) and anti-mouse (1:40000, GE Health, code number NA931). All primary antibodies were diluted in 1X TBS-0.1% Tween 20 (TBST) containing 5% BSA and 0.02% sodium azide; all secondary antibodies were diluted in 1X TBST containing 1% BSA. Bands were quantified through Quantity One software (Biorad). Protein phosphorylation was normalized to the total amount of the corresponding protein (for each sample the ratio between the phosphorylated band and the total protein was calculated) and the result is shown in the graphic relatively to the control samples.

### Statistical analysis

Statistical analyses were performed using Prism 5.0 for Windows (GraphPad software). For statistical evaluations of behavioral tests, One-way ANOVA followed by a Bonferroni *post-hoc* for multiple comparisons was used. For cellular and molecular analyses, statistical comparisons were conducted by two-tail unpaired Student's *t*-test. In all cases, significance levels were set at *p* ≤ 0.05. Data are expressed as mean ± SEM. Behavioral tests were carried out on 10 different mice in each group. Cell counts and DG volumes are derived from at least three different animals per group.

## Results

### Behavioral impairments in mice exposed to chronic/subchronic paint thinner inhalation

**Figure 1:**
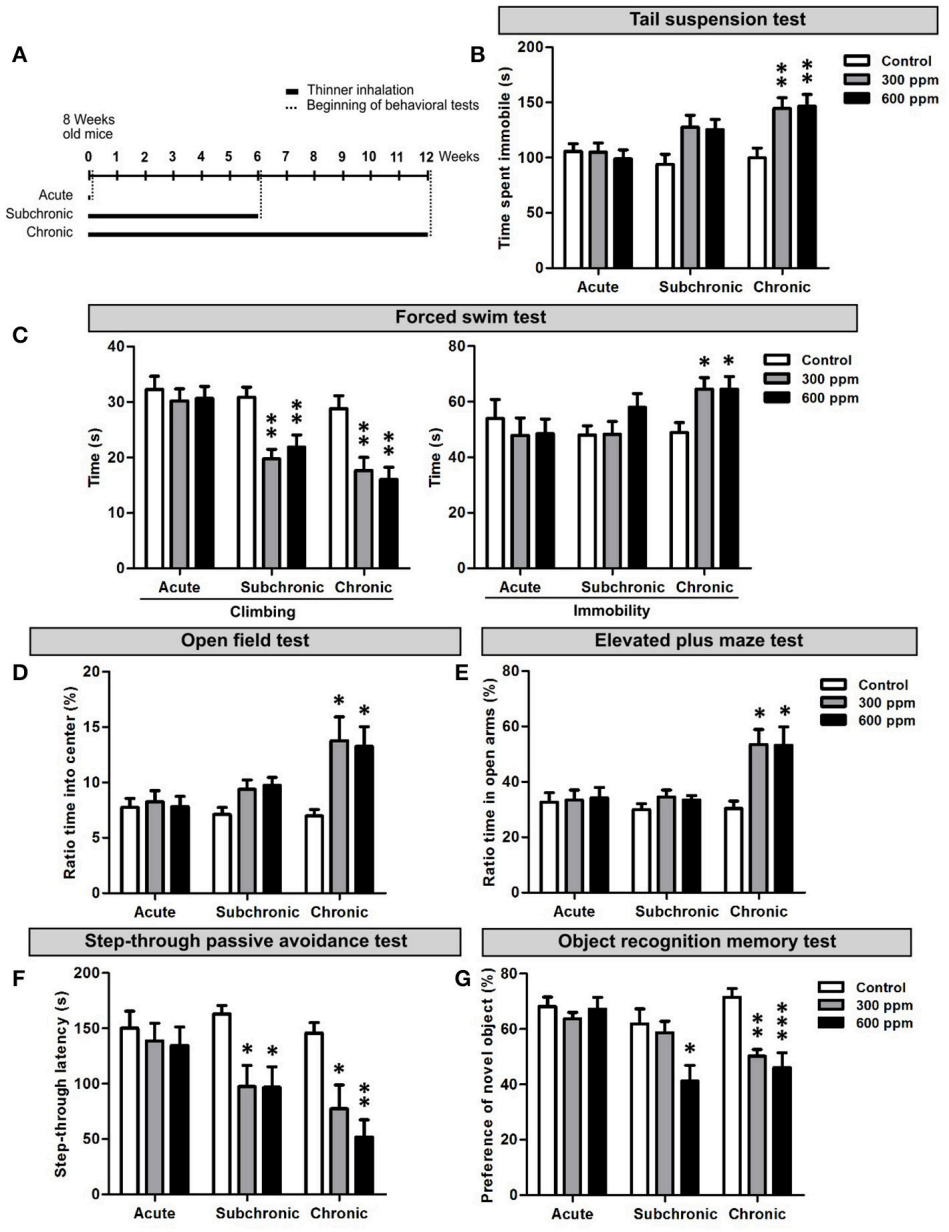
Behavioral dysfunctions in adult mice following thinner inhalation. **(A)** Experimental design: mice were exposed for two sessions/day 8 h apart for 1 day (acute), 6 weeks (subchronic), or 12 weeks (chronic); treatments were followed by the behavioral tests, conducted starting from 24 h after the last inhalation. **(B)** Time of immobility measured during tail suspension test. **(C)** Climbing and immobility time calculated during forced swim test. **(D)** Ratio of time spent into center of the arena in the open field test over the total time spent in the arena. **(E)** Ratio of time spent in the open arms over the total time spent in both the open and enclosed arms in the elevated plus maze test. **(F)** step-through latency measured in step-through inhibitory avoidance test. **(G)** Ratio of time spent exploring the novel object over the total time spent exploring both objects. Error bars indicate SEM (*n* = 10 per group). Bonferroni *post-hoc*, ^*^p < 0.05, ^**^p < 0.01, and ^***^p < 0.001 refer to the control vs. treated groups comparison.

To analyze the effect of paint thinner inhalation on adult mice we first performed a set of behavioral tests assessing depression-related responses, anxiety, learning and memory functions. The behavioral analyses started from the day after the last thinner exposure. The experimental design included acute (two exposures 8 h apart in the same day), subchronic (two exposures/day, repeated for 6 weeks), and chronic (two exposures/day, repeated for 12 weeks) treatments (Figure 1A). For each experimental set, two thinner concentrations (i.e., 300 and 600 ppm) were tested and the results compared to control conditions (Figure 1A; summary of data and statistical analyses are presented as Supplementary Table 2).

To assess depression-like behaviors we performed the TST and FST. In the TST we found a statistically significant increase in the time spent in immobility in chronically treated mice [One-way ANOVA, *F*_(2, 27)_ = 7.33, *p* = 0.003; Figure 1B]. Both thinner concentrations used induced similar increased immobility compared to control mice (Figure 1B). However, although subchronically treated mice showed longer immobility time in average and One-way ANOVA indicates an effect of treatment [*F*_(2, 27)_ = 3.70, *p* = 0.04], Bonferroni *post-hoc* comparison reveals no significant differences among groups (Supplementary Table 2). No differences were observed in the time spent in immobility following acute treatment [One-way ANOVA, *F*_(2, 27)_ = 0.23, *p* = 0.79]. In the FST, similarly to TST, the time of immobility increased significantly only after chronic treatments, with no dose effect [One-way ANOVA, *F*_(2, 27)_ = 4.71, *p* = 0.018; Figures 1C]. On the other hand, a statistically significant decrease in the time spent for climbing behavior was observed in mice treated with either 300 or 600 ppm of thinner in subchronic [One-way ANOVA, *F*_(2, 27)_ = 9.43, *p* = 0.001; Figure 1C] and chronic [One-way ANOVA, *F*_(2, 27)_ = 9.07, *p* = 0.001; Figure 1C], but not acute [One-way ANOVA, *F*_(2, 27)_ = 0.24, *p* = 0.79] treatments (Supplementary Table 2). All together, these data suggest that signs of depression-like behaviors start to emerge following 6 weeks of thinner treatment and strengthen in longer exposure protocols (i.e., 12 weeks).

The OFT and EPMT were applied to assess anxiety-related behaviors. In the OFT, mice chronically exposed to either 300 or 600 ppm of thinner spent significantly more time in the center of the open field [One-way ANOVA, *F*_(2, 27)_ = 5.22, *p* = 0.012; Figures 1D], with no dose effect (Supplementary Table 2). Similarly, chronic treatment also increased the time spent in the open arms of the elevated plus maze [One-way ANOVA, *F*_(2, 27)_ = 6.56, *p* = 0.005; Figure 1E], with comparable effects for the two thinner concentrations. The subchronic treatment had no effect in the EPMT [One-way ANOVA, *F*_(2, 27)_ = 1.26, *p* = 0.301; Figure 1E], while increased the time spent in the center of the arena in the OFT [One-way ANOVA, *F*_(2, 27)_ = 3.66, *p* = 0.039; Figure 1D]. However, here Bonferroni *post-hoc* comparison between groups does not show statistically significant difference (Supplementary Table 2). Finally, no change in anxiety-related behavior was observed after acute treatment in both OFT [One-way ANOVA, *F*_(2, 27)_ = 0.09, *p* = 0.910; Figure 1D] and EPMT [One-way ANOVA, *F*_(2, 27)_ = 0.06, *p* = 0.956], indicating that a clear anxiolytic effect only occurs after chronic thinner exposure.

Next, we assessed the consequences of thinner exposure on learning and memory functions by step-through passive avoidance test (SPAT) and object recognition memory test (ORMT) (Figures 1F,G). In the SPAT both subchronic and chronic treatments induced a robust and significant shortening in the step-through latency time compared to controls, with no dose effect [One-way ANOVA, subchronic: *F*_(2, 27)_ = 5.57, *p* = 0.009; chronic: *F*_(2, 27)_ = 8.89, *p* = 0.001; Figure 1F and Supplementary Table 2]. No change in the step-through latency time was observed following acute treatment [One-way ANOVA, *F*_(2, 27)_ = 0.26, *p* = 0.773]. Similarly, in the ORMT, mice acutely exposed to thinner spent significantly more time exploring the novel object compared to the familiar one, behaving as control mice [One-way ANOVA, *F*_(2, 27)_ = 0.46, *p* = 0.636; Figure 1G]. Following chronic treatment, mice spent nearly equal time exploring the familiar and novel objects (Figure 1G), with a statistically significant difference when compared to control mice [One-way ANOVA, *F*_(2, 27)_ = 12.62, *p* = 0.001; Supplementary Table 2], which spent more time exploring the novel object. Interestingly, the subchronic treatment also induced a significant effect in the ORMT [One-way ANOVA, *F*_(2, 27)_ = 4.67, *p* = 0.018], although limited to the higher concentration used (Figure 1G and Supplementary Table 2). Overall, our findings indicate that long term repeated exposure to thinner negatively impacts on cognitive functions, whereas acute thinner inhalation has no major effect on the tested behavioral performances.

### Chronic paint thinner exposure affects the proliferative activity of neural stem/progenitor cells in the hippocampal DG

**Figure 2:**
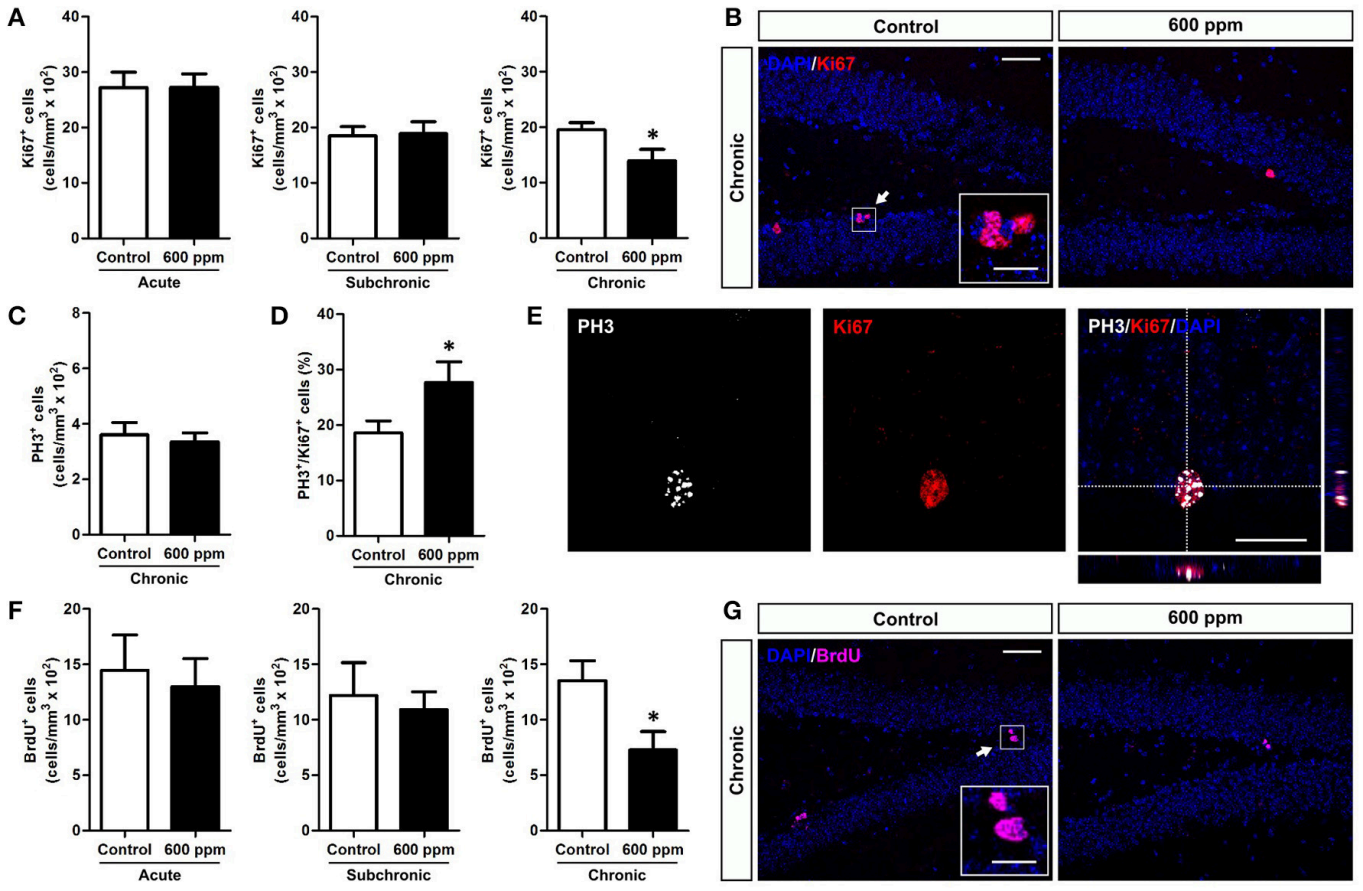
Chronic thinner exposure reduces neural stem/progenitor cell proliferation in the adult DG. **(A**) Mean of cell density of Ki67^+^ cells within the hippocampal DG following acute, subchronic and chronic thinner exposure. **(B**) Representative confocal images of coronal DG sections with DAPI nuclear counterstain (blue) showing Ki67^+^ cells in red, in control and 600 ppm-treated animals following chronic treatment. **(C**) Mean of cell density of PH3^+^ cells within the hippocampal DG following chronic thinner exposure. **(D)** Percentage of double-labeled PH3^+^/Ki-67^+^ cells among Ki-67^+^ cells following chronic thinner exposure and in control animals. **(E)** Representative confocal images of the DG showing a nucleus double stained for Ki-67 (red) and PH3 (white) with DAPI counterstaining in blue. **(F)** Mean of cell density of BrdU^+^ cells within the hippocampal DG following acute, subchronic, and chronic thinner exposure (analysis at 24 h after BrdU injection). **(G)** Representative confocal images showing BrdU^+^ cells (magenta) with DAPI counterstaining in blue, in control and 600 ppm-treated animals following chronic treatment. Error bars indicate SEM (*n* = 4–5 per group). Student's *t*-test, ^*^p < 0.05 refers to the control vs. treated groups' comparison. Scale bar: 50 μm in **(B,G)**; 15 μm in insets; 25 μm in **(E)**.

A growing number of data correlate emotional and cognitive abnormalities with dysfunction of the hippocampal dentate gyrus (DG), including impaired generation of new DG neurons (Yun et al., 2016). Indeed, the adult DG is endowed with continuous generation of new neurons that play a role in memory, mood, pattern separation and reward (Jessberger et al., 2009; Noonan et al., 2010; Denny et al., 2014; Hill et al., 2015; Danielson et al., 2016). Adult born neurons originate from proliferation of neural stem/progenitor cells within the sub-granular zone (SGZ) of the DG (Aimone et al., 2014). Notably, genesis and integration of new neurons in this region are dynamically regulated by both environmental and physiological factors (Van Praag et al., 1999; Kempermann, 2011, 2015). To assess the impact of thinner inhalation on DG neurogenesis in adult mice, we first evaluated the number of cells expressing Ki67, an endogenous marker of cell proliferation that labels SGZ stem/progenitor cells in the cell cycle but not in the resting phase (von Bohlen und Halbach, 2011), in animal treated with 600 ppm compared to controls. Our data show that chronic exposure to thinner, but not subchronic or acute treatment, reduces the density of Ki67^+^ cells, indicating impaired DG stem/progenitor proliferation (Student's *t*-test, *p* < 0.05; Figures 2A,B). Importantly, analysis of the DG granule cell volume among the different experimental groups revealed no statistically significant differences between treated (T) and control (C) animals (acute: C = 0.336 ± 0.023 mm^3^, T = 0.351 ± 0.010 mm^3^; subchronic: C = 0.352 ± 0.045 mm^3^; T = 0.356 ± 0.029 mm^3^; chronic: C = 0.418 ± 0.069 mm^3^; T = 0.407 ± 0.035 mm^3^; Student's *t*-test, all at *p* > 0.05).

Interestingly, no changes in the number of cells positive for the mitotic marker PH3 (von Bohlen und Halbach, 2011) were found within the SGZ of chronically treated mice (Student's *t*-test, *p* = 0.33; Figure 2C), and the percentage of Ki67 positive cells co-expressing PH3 showed an increase in chronically treated compared to control mice (Student's *t*-test, *p* < 0.05; Figures 2D,E). On the whole, these data suggest a possible shortening of the cell cycle of DG progenitors. To further analyze proliferation of neural stem/progenitor cells we applied a single pulse BrdU protocol in which animals received an injection of BrdU the day after the last thinner exposure and were perfused 24 h later (Figures 2F,G). After chronic treatment exposure we found decreased density of newborn BrdU^+^ cells by 46% (Student's *t*-test, *p* < 0.05), indicating a net reduction in progenitor proliferation. In line with the Ki67 data, no difference was observed in the density of BrdU^+^ cells in acutely and subchronically treated mice (Figure 2F).

### Chronic/subchronic thinner exposure impairs survival of DG newborn neurons

**Figure 3:**
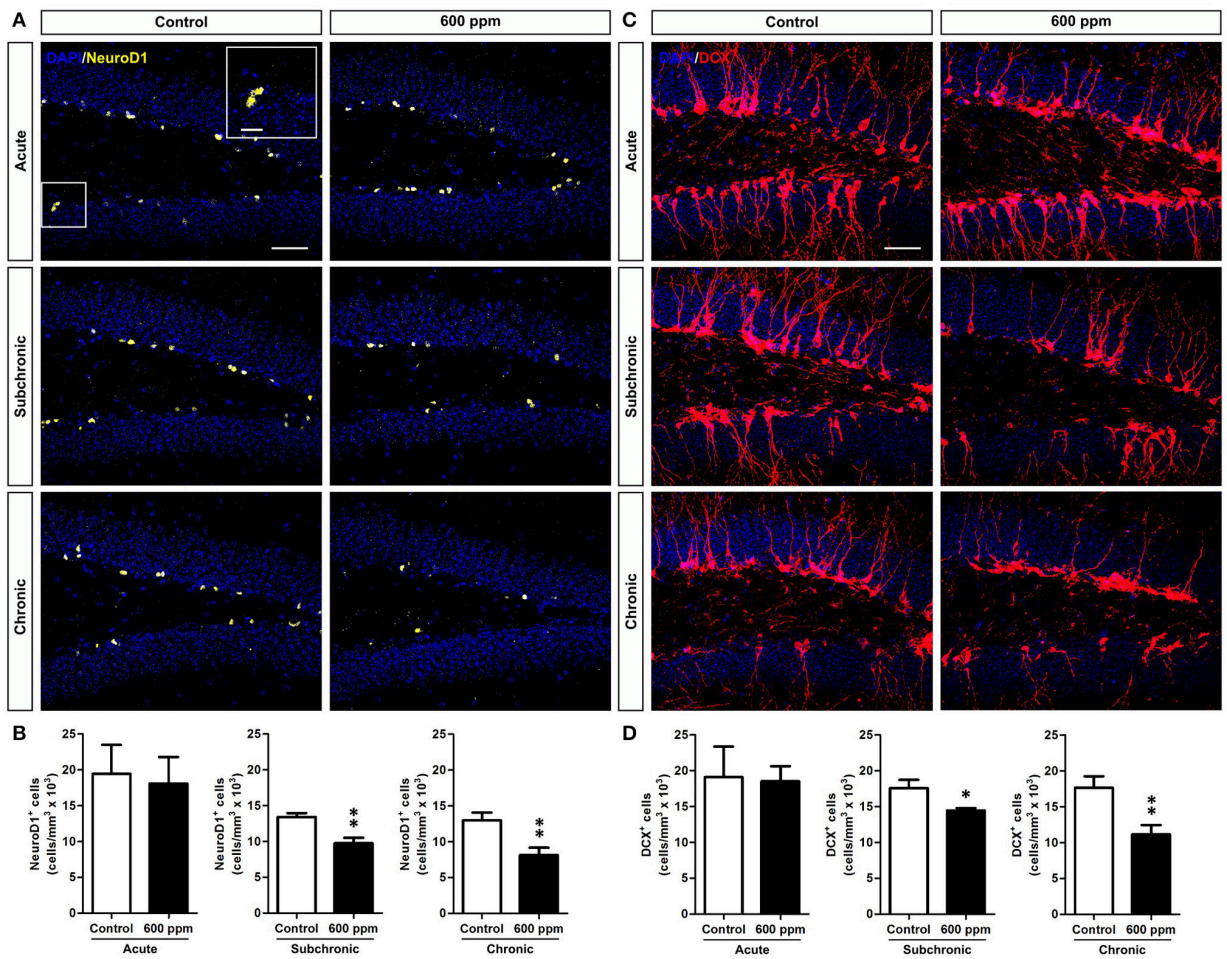
Subchronic and chronic thinner exposures affect neurogenesis in the adult DG. **(A)** confocal images of DG sections stained in yellow for NeuroD1 with DAPI counterstaining (in blue) in control and 600 ppm-treated mice following acute, subchronic and chronic inhalation. **(B)** Mean of cell density of NeuroD1^+^ neurons within the hippocampal DG in acute, subchronic and chronic groups. **(C)** confocal images of DG sections stained in red for DCX with DAPI counterstaining (in blue) in control and 600 ppm-treated mice following acute, subchronic and chronic inhalation. **(D)** DCX^+^ cell density within the hippocampal DG in acute, subchronic, and chronic groups. Error bars indicate SEM (*n* = 5 per group). Student's *t*-test, ^*^p < 0.05 and ^**^p < 0.01 refer to the control vs. treated groups' comparison. Scale bar: 50 μm in **(A,C)**.

We next sought to assess the impact of thinner exposure on DG newly generated neurons. To this aim we used NeuroD1 as a marker to label early cells of the neuronal lineage (i.e., neural committed progenitors, neuroblasts, and immature neurons; Gao et al., 2009), and doublecortin (DCX), which labels immature neurons in the SGZ and granule cell layer (GCL) of the DG (von Bohlen und Halbach, 2011). We found a net reduction in NeuroD1^+^ cells in chronically treated mice (Student's *t*-test, *p* < 0.01; Figures 3A,B). Interestingly enough, a statistically significant decrease was also observed following subchronic treatment (Student's *t*-test, *p* < 0.01; Figures 3A,B), while no changes were found in acute treatment (Student's *t*-test, *p* = 0.40; Figures 3A,B). Quantification of DCX-immunostaining confirmed the same pattern, with both subchronic and chronic treatments effective in reducing the amount of neuronal progenitors and newborn neurons (Student's *t*-test, *p* < 0.05; *p* < 0.01, respectively; Figures 3C,D), and no effect for acute treatment (Student's *t*-test, *p* = 0.44).

**Figure 4:**
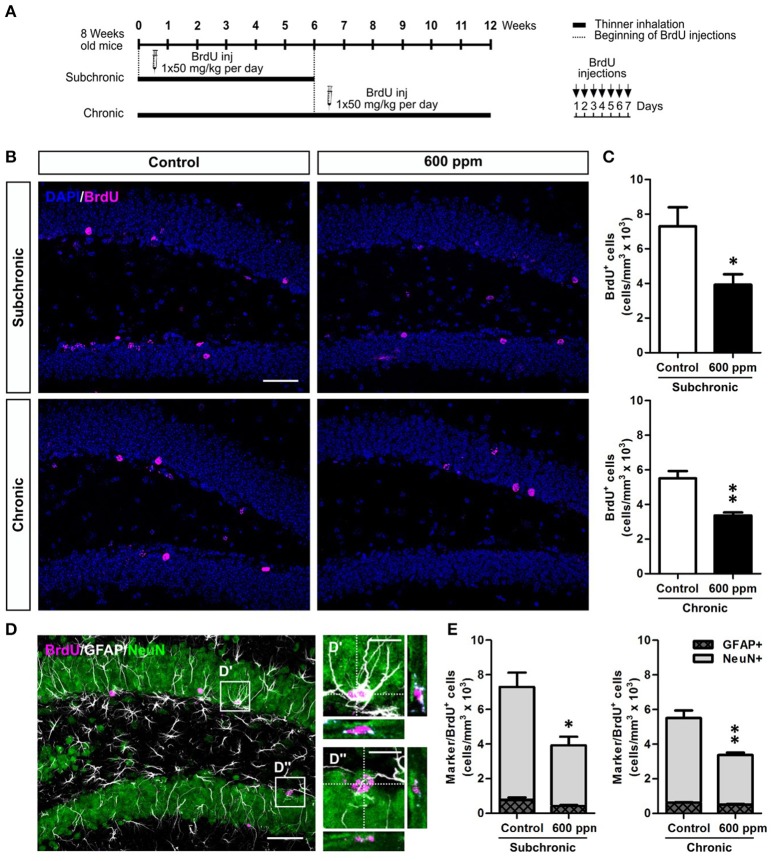
Subchronic and chronic thinner exposures impair survival of DG newborn neurons without altering the balance between neurogenesis and astrogliogenesis in the adult DG. **(A)** Experimental design: controls and 600 ppm-treated mice received multiple BrdU injections over 1 week, starting from the beginning of the subchronic treatment and on week 6 for the chronic treatment; animals were analyzed at 5 weeks survival post-BrdU injection in both cases. **(B)** Representative confocal images of the DG stained for BrdU (magenta), with DAPI counterstaining (in blue) in control and 600 ppm-treated mice (subchronic and chronic treatments). **(C)** BrdU^+^ cell density in subchronic and chronic groups. **(D)** Representative confocal image of triple staining for BrdU (magenta), GFAP (white), and NeuN (green). Insets in **(D)** are displayed at higher magnification with re-slicing in **(D',D”)**, and show a cell double-labeled for BrdU and GFAP **(D')** and a cell double-labeled for BrdU and NeuN **(D”)**. **(E)** Cell density of double labeled cells for BrdU/GFAP and BrdU/NeuN among the BrdU^+^ cell population in subchronic and chronic groups. Error bars indicate SEM (*n* = 3–4 per group). Student's *t*-test, ^*^p < 0.05 and ^**^p < 0.01 refer to the control vs. treated groups' comparison. Scale bar: 50 μm in **(B,D)**; 15 μm in insets **(D',D”)**.

While a decreased number of newborn neurons in chronically treated mice could be the direct consequence of the reduced stem/progenitor proliferation described above (Figure 2), the results in the subchronic group clearly point to a survival effect that could also partly contribute to the outcome of the chronic treatment. To further evaluate the survival of neurons generated under thinner treatment we labeled a pool of newborn cells by multiple BrdU injections over 1 week, starting from the beginning of the subchronic treatment and on week 6 for the chronic treatment (Figure 4A). In both cases, animals were analyzed at 5 weeks post-BrdU injections. Our data show a significant decrease in the total number of BrdU^+^ cells both in the subchronic (Student's *t*-test, *p* < 0.05; Figures 4B,C) and in the chronic (Student's *t*-test, *p* < 0.01; Figures 4B,C) groups, further supporting a defective survival of adult born neurons. By triple staining for BrdU, the late post-mitotic neuron marker NeuN and the glial marker GFAP (Figures 4D–D”; von Bohlen und Halbach, 2011) we found that, as for control animals, also in the treated groups the large majority of BrdU^+^ cells were double positive for NeuN, while only a minority were GFAP+ with a typical astrocytic-like morphology (Figure 4E). Moreover, no radial-glial like cells were observed among the BrdU+/GFAP+ population. These results indicate that the proportion between neurogenesis and astrogliogenesis is not altered by thinner exposure.

**Figure 5:**
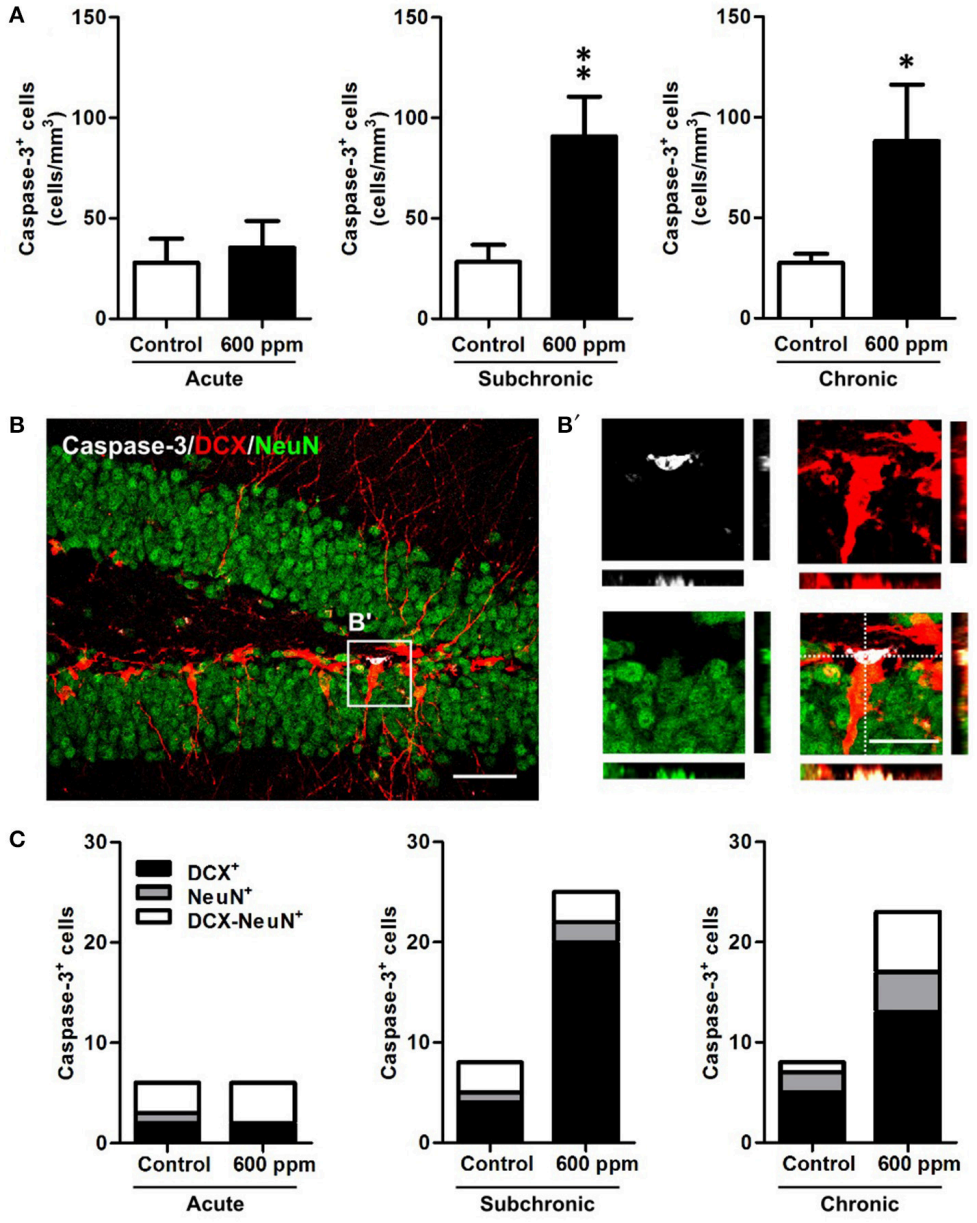
Enhanced apoptosis of DG newborn neurons following subchronic and chronic thinner inhalation. **(A)** Caspase-3^+^ cell density within the hippocampal DG in acute, subchronic and chronic groups in control and 600 ppm-treated mice. **(B)** Representative confocal image of the DG of a chronically treated animal labeled for Caspase-3 (white), DCX (red), and NeuN (green). Inset in **(B)** is displayed at higher magnification with re-slicing in **(B')** and shows a caspase-3/DCX double positive cell, negative for NeuN. **(C)** Relative proportions of DCX^+^, NeuN^+^, and DCX/NeuN^+^ cells among the total number of Caspase-3^+^ cells in acute, subchronic and chronic groups. Error bars indicate SEM (*n* = 5 per group). Student's *t*-test, ^*^p < 0.05 and ^**^p < 0.01 refer to the control vs. treated groups' comparison. Scale bar: 50 μm in **(B)**; 15 μm in insets **(B')**.

Next, to examine whether thinner inhalation treatments trigger programmed cell death, we evaluated the number of apoptotic cells in the DG by counting the cells expressing caspase-3 (Figure 5). A 3-fold significant increase in DG apoptotic cells was found in both subchronic and chronic groups compared to controls (Student's *t*-test, *p* < 0.01; *p* < 0.05, respectively), while no differences were observed after acute thinner inhalation (Student's *t*-test, *p* = 0.34). All these apoptotic cells belong to the neuronal lineage, being either DCX^+^, NeuN^+^, or DCX/NeuN double^+^ (Figures 5B,C). By comparing the caspase-3^+^ cells that were positive for each marker or co-express both markers in treated vs. control mice (Figure 5C), we show that the amount of caspase-3^+^ cells expressing NeuN, but negative for DCX, remains nearly unaltered in treated mice, while an expansion was observed in caspase-3/DCX double^+^ cells (Figure 5C), suggesting that increased cell death due to thinner inhalation mostly involves newly born neuroblasts and immature neurons.

### Molecular correlates of behavioral and cellular alterations in mice exposed to chronic/subchronic paint thinner inhalation

**Figure 6:**
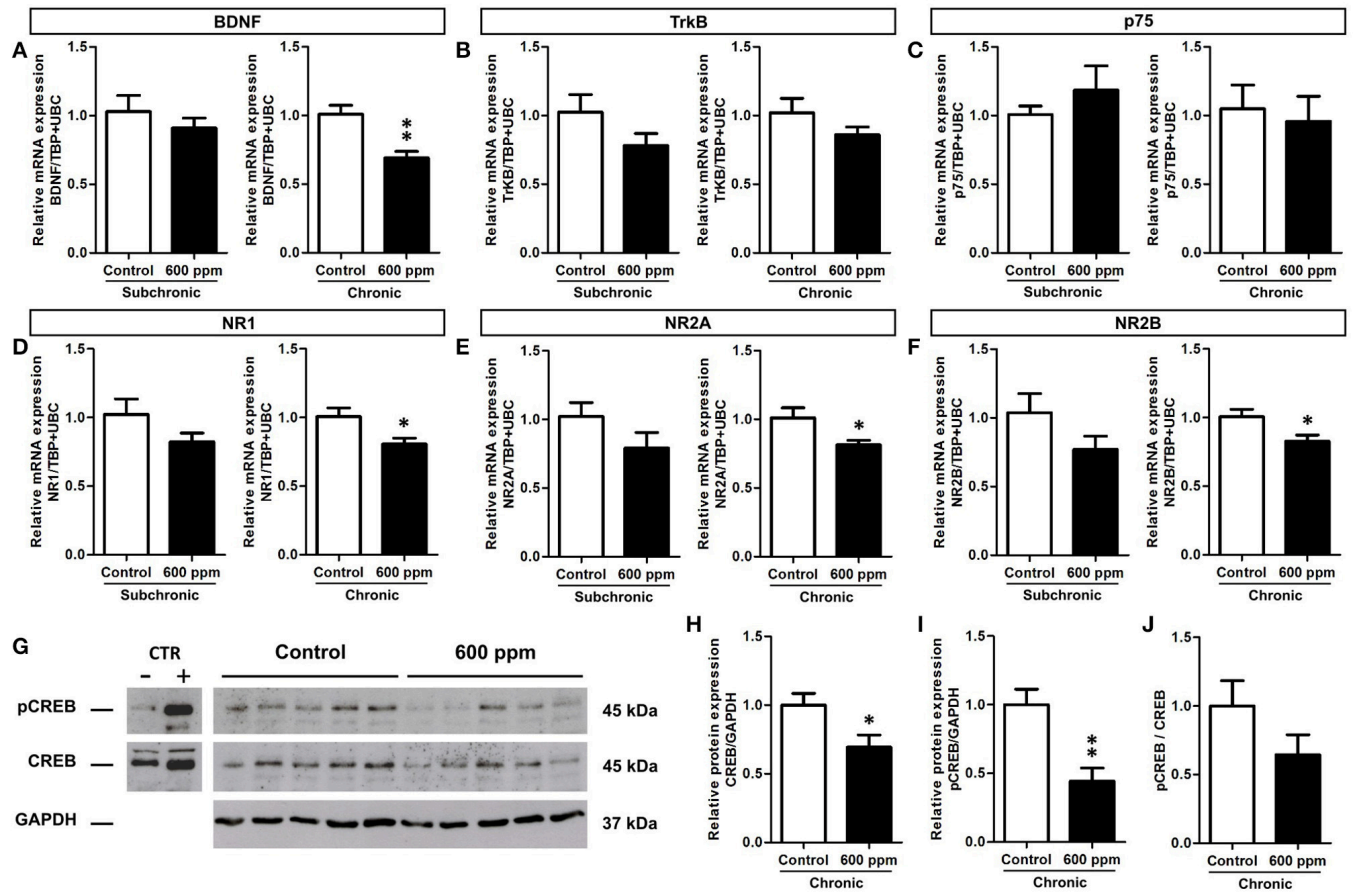
Reduced BDNF, NMDA receptor subunits and CREB protein expression/phosphorylation in adult hippocampus following chronic thinner inhalation. **(A–F)** Relative mRNA expression of BDNF **(A)**, TrkB **(B)**, p75 **(C)**, NR1 **(D)**, NR2A **(E)**, and NR2B **(F)** in subchronic and chronic groups. The relative quantifications (expressed as −2^ΔΔ*Ct*^) were obtained by quantitative real-time PCR and data were normalized to the geometric average of two endogenous housekeeping genes (TBP and UbC). **(G)** Western blot panels showing pCREB and CREB protein levels in hippocampus of chronically exposed mice to 0 and 600 ppm of thinner. As negative and positive control of CREB phosphorylation, neuronal progenitors expressing ErbB4 (Fornasari et al., 2016), untreated or treated for 15 min with 10 nM soluble Neuregulin1, were used. Molecular mass standards are expressed in kilodaltons (kDa). **(H–J)** The protein levels of CREB **(H)** and pCREB **(I)** determined by using GAPDH as an internal control in chronic groups. Each data point was normalized to the control (0 ppm). CREB phosphorylation was normalized also to the total amount of the protein (for each sample the ratio between the phosphorylated band and the total protein was calculated) and the results are shown in the graphic **(J)**. Error bars indicate SEM (*n* = 5 per group). Student's *t*-test, ^*^p < 0.05 and ^**^p < 0.01 refer to the control vs. treated groups' comparison.

Proliferation and survival of neural stem/progenitor cells and newly born neurons are tightly regulated within the hippocampal neurogenic niche (Kempermann, 2015). In this context, we chose to analyze the expression of the brain derived neurotrophic factor (BDNF) and the glutamate N-Methyl-D- aspartic acid receptor (NMDAr), that play well established cooperative regulatory roles in adult hippocampal neuroplasticity (Duman and Voleti, 2012; Begni et al., 2017). We first analyzed by quantitative real-time PCR the transcripts for BDNF in the hippocampi of mice following subchronic and chronic thinner inhalation (Figure 6A). A significant decrease in the BDNF mRNA expression was evident in thinner-exposed mice after chronic inhalation (Student's *t*-test, *p* = 0.004; Figure 6A), while no statistically significant differences were found in the subchronic group (Student's *t*-test, *p* = 0.41). In addition, no changes were observed in the mRNA expression of the BDNF receptors TrkB and p75 (Student's *t*-test, TrkB: chronic p = 0.15; subchronic *p* = 0.21; p75: chronic *p* = 0.37; subchronic *p* = 0.72; Figures 6B,C). We next investigated the expression of NMDAr subunits NR1, NR2A, and NR2B (Figures 6D–F). All subunits showed significantly decreased levels of mRNA expression in mice chronically exposed to thinner, as compared to control mice (Student's *t*-test, NR1: *p* = 0.03; NR2A: *p* = 0.04; NR2B: *p* = 0.04; Figures 6D–F). Although the relative mRNA expression following subchronic treatment appeared to be lower in average compared to controls, here the differences did not reach statistically significant values (Student's *t*-test, all at *p* > 0.05).

The cAMP response element-binding protein (CREB) is one of the best studied transcription factors acting downstream of BDNF signaling (Reichardt, 2006) and regulating both BDNF and NMDAr expression (Carlezon et al., 2005). We thus focused on CREB protein levels and phosphorylation to further investigate the effect of chronic paint thinner exposure on hippocampal function (Figure 6G). Notably, in treated mice we found a 25% reduction in CREB protein expression (Student's *t*-test, *p* = 0.04; Figure 6H) and a nearly 50% decrease in the level of its phosphorylated form (Student's *t*-test, *p* = 0.005; Figure 6I). Moreover, the pCREB/CREB average ratio was higher in control than in treated mice, although this difference does not reach statistical significance (Figure 6J, Student's *t*-test, *p* = 0.17), it suggests that thinner treatment negatively influences CREB signaling not only by reducing its expression but possibly also inhibiting its phosphorylation. The observed changes in mRNA expression for BDNF and NMDAr, together with the reduction in CREB expression/phosphorylation (Figures 6H–J) can underlie altered hippocampal functions, including the observed alteration in DG neurogenesis and behavior following long term thinner inhalation.

## Discussion

Abuse of inhalants is a worldwide issue, with a higher incidence among poor/marginalized communities, affecting people of all ages and leading to significant health and psychosocial outcomes. Inhalants comprise different groups of chemicals, including volatile anesthetics, nitrous oxide, alkyl nitrites, and volatile solvents, the last being the most commonly abused class of inhalants (Beckley and Woodward, 2013). While toluene is by large the solvent that received much attention in basic research studies (Cruz et al., 2014), here, to reproduce the synergistic effects of the mixture of different aromatic hydrocarbons found in common sources of abused solvents (e.g., spray, paint thinner, petroleum), we exposed adult mice to paint thinner inhalation following a previously established experimental design (Fifel). We characterized the effects of thinner exposure on mice at the behavioral, cellular and molecular levels, focusing on the hippocampus, and particularly on the dentate gyrus (DG). Indeed, neurogenesis is maintained in the adult DG where it plays an important role in hippocampal-mediated functions (Yun et al., 2016). In spite of the large body of literature supporting dysfunctions of cortical and subcortical brain regions (e.g., nucleus accumbens and ventral tegmental area) as a consequence of volatile solvents exposure (Beckley and Woodward, 2013), data on the effects of volatiles on the hippocampus are only fragmentary.

The effects of acute (i.e., single-day exposure) and prolonged (i.e., subchronic and chronic) thinner inhalation on hippocampal-related functions in adult mice were initially assessed through behavioral tests for depression (i.e., TST and FST), anxiety (i.e., OFT and EPMT), learning and memory (i.e., SPAT and ORT). While previous studies have shown that acute inhalant exposure on adult animals results in various alterations in hippocampal-related behaviors immediately after treatment (Páez-Martínez et al., 2003; Lo et al., 2009; Fifel et al., 2014), our data clearly showed that 24 h after acute thinner exposure none of the tested behaviors were affected, suggesting no lasting change in the hippocampal system. Accordingly, we reported normal levels of progenitor proliferation (Ki67^+^ and BrdU^+^ cell densities) and newborn neuron generation (NeuroD1^+^ and DCX^+^ cell densities) in the DG of acutely treated mice.

In contrast, in a previous study, reduced Ki67^+^ and DCX^+^ cells in hippocampal DG were observed in mice acutely treated with an intraperitoneal injection of toluene (500 mg/kg) and analyzed 24 h and 4 days later (Seo et al., 2010), supporting in this case impaired neurogenesis after acute treatment. Altered DG neurogenesis in these mice was associated to a depression-like behavior and impaired memory functions assessed in the TST, FST, contextual fear conditioning and ORT, 24 h and 4 days after treatment (Seo et al., 2010). However, at such short survival time a direct causal link between altered neurogenesis and change in hippocampal-related behavior is unlikely, considering the time needed for a newborn neuron to functionally integrate into the hippocampal circuit (i.e., 3–4 weeks; Aimone et al., 2014).

Decreased hippocampal neurogenesis was also found in rats, following acute high-level toluene inhalation (i.e., exposure to 7000 ppm for 4 h; Yoon et al., 2015). Although we cannot exclude that the use of paint thinner instead of toluene could possibly partly justify the absence of a neurotoxic effect in the acute treatment performed in our study, the route of administration and most importantly the dose/duration of inhalant exposure, are likely to play a major role in the effect of inhalants and to explain the observed discrepancy.

Accordingly, in the subchronic treatment, in which mice were exposed to thinner daily for a period of 6 weeks, signs of depressive-like behavior start to emerge in parallel to significant alterations in memory/learning related functions as assessed in the SPAT and ORT. These findings confirm our previous data (Fifel et al., 2014) and are in agreement with another study in rats showing cognitive deficits in the SPAT and Morris water maze following a 45 days-long thinner exposure treatment (Baydas et al., 2005a,b). In line with these behavioral data, adult DG neurogenesis significantly decreased in the subchronic group where we observed a reduction in both neural committed progenitors/neuroblasts (i.e., NeuroD1^+^ cells) and immature neurons (i.e., NeuroD1 and DCX^+^ cells), as well as a deficit in DG newborn cell survival (i.e., BrdU long term study) compared to controls. Moreover, we demonstrated that subchronic thinner inhalation selectively triggers programmed cell death in immature newborn neurons (i.e., DCX^+^ cells) having no effects on hippocampal mature granule cell survival (i.e., NeuN^+^ cells) and on the proliferative capacity of DG progenitors. This highlight a major difference with a previous study (Seo et al., 2010), in which inhibition of hippocampal neurogenesis occurs without significant induction of apoptosis and is mainly associated to decreased proliferation in the DG upon a single high-dose injection of toluene. Thus, supporting the involvement of distinct cellular/molecular mechanisms underlying the detrimental effects of solvent exposure in the two experimental sets, with our design being a more reliable model for inhalant abuse.

Notably, we found that a chronic treatment, implying 12 weeks of daily thinner inhalation, induced depression-like behaviors (i.e., TST and FST) and significant impairments in learning/memory functions (i.e., SPAT and ORT), suggesting a progressive worsening of the inhalant effects with time of exposure. In addition, the results revealed an anxiolytic effect (i.e., OFT and EPMT) due to chronic thinner exposure. Considerable evidence indicates that anxiety and memory are closely linked processes (Beuzen and Belzung, 1995; Ribeiro et al., 1999; Podhorna and Brown, 2002); the mean anxiety level is known to facilitate efficient learning on a conditioned passive avoidance reflex, while high and low levels lead to suppression of memory. Therefore, based on our results, we cannot exclude a possible impact of the anxiolytic effect on the associative learning alterations observed in treated mice. Although to our knowledge this is the first study assessing such a prolonged period of inhalant exposure on animal behaviors, several clinical reports indicate that chronic inhalant exposure in humans produces an altered personality with significant signs of depression, anxiety, and cognitive impairment (Maruff et al., 1998; Cairney et al., 2002; Chouanière et al., 2002; Grant et al., 2004; Perron and Howard, 2009). In line with the subchronic group, also chronically treated mice showed impaired adult DG neurogenesis. Moreover, in addition to the reduced survival of DG newborn neurons, in this group we also observed a significant decrease in DG progenitor proliferation. Further studies are needed to clarify possible alterations induced by chronic thinner inhalation on cell cycle dynamics that might eventually produce aberrant neural stem/progenitor cell division with long-term effect on behavior. Additionally, we found that the expression levels of BDNF and NMDA receptors, which are critically involved in regulating adult neurogenesis, synaptic plasticity, and memory formation (Aimone et al., 2014; Begni et al., 2017), are downregulated following chronic treatment. Specifically, chronic inhalant exposure induces reduced BDNF expression, without changing the expression levels of its receptors TrkB and P75. However, while no significant changes were found in the subchronic group, BDNF and TrkB were previously reported to be overexpressed in mice exposed to toluene (i.e., 500 ppm) for 6 h a day in subchronic treatments (Win-Shwe and Fujimori, 2010). On the same line, although our data clearly indicate a downregulation in NMDA receptor subunits NR1, NR2A, and NR2B following chronic treatment, previous studies reported increased expression of NMDA NR2B subunit in mice chronically exposed to low (50 ppm) toluene concentration (Ahmed et al., 2007). A synergistic effect of the mixture of substances composing paint thinner might underlie the observed reduction in hippocampal-plasticity related molecules that is not observed for toluene exposure alone. Alternatively, these contradictory results could be due to a dose-dependent effect. Activation of postsynaptic NMDA receptor triggers complex downstream signaling events, including cAMP response-element binding protein (CREB)-dependent gene transcription, which is a critical regulator of essential developmental steps in adult neurogenesis and memory formation (Merz et al., 2011). Moreover, CREB acts downstream of BDNF signaling (Reichardt, 2006) and regulate in turn both BDNF and NMDA receptor expression (Carlezon et al., 2005). Interestingly, we showed that chronic treatment also reduces CREB expression/phosphorylation.

Taken together, our findings add new insights in understanding the neurobiological basis for solvent abuse, supporting the notion that adult DG neurogenesis and more generally hippocampal neuroplasticity are selective targets of the negative effects induced by prolonged inhalant exposure and play a role in the behavioral dysfunctions associated to inhalant abuse.

## Author contributions

HM, SB-M, and MB: designed and planned paint thinner treatments and behavioral experiments; HM, SB, and SDM: designed and planned BrdU experiments, immunofluorescence analyses and cell counting; HM, GG, and IP: designed and planned Real-time PCR and Western Blot analyses; HM: performed all experiments and analyses; HM, SB-M, MB, and SDM: wrote the paper; SB-M and SDM: jointly supervised the research; All authors agree to be accountable for the content of the work.

### Conflict of interest statement

The authors declare that the research was conducted in the absence of any commercial or financial relationships that could be construed as a potential conflict of interest.
