# Supplementary material for: Decreased Hippocampal Neuroplasticity and Behavioral Impairment in an Animal Model of Inhalant Abuse
Source: Front Neurosci. 2018 Feb 6;12:35. doi: 10.3389/fnins.2018.00035 (PMC5810293; doi:10.3389/fnins.2018.00035)
Supplement: Supplementary file 1 [file Table1.DOCX]

# Supplementary Table 1

| Gene | Accession number | Forward primer (5'-3') | Reverse primer (5'-3') | Amplicon size | Primer efficiency |
| --- | --- | --- | --- | --- | --- |
| BDNF | NM_001285416.1 | GCGTGTGTGACAGTATTAGCGAGTG | CAGTTGGCCTTTGGATACCGGG | 116 | 95 |
| TrkB | NM_001282961.1 | GAAAAACAGCAACCTGCGGCAC | GAACGGATTACCCGTCAGGATCAGG | 115 | 101 |
| p75 | NM_033217.3 | CCGAATGCGAGGAGATCCCTGG | CCTGGGTGCTGGGTGTTGTG | 86 | 81 |
| NR1 | NM_001177657.2 | CGTCCTGGGGCTGACTACCC | GCTGGACTGGTGGGAGTAGGG | 97 | 105 |
| NR2A | NM_008170.2 | GACCCACTGACTGAGACCTGCG | CCCCTTGCAGCACTTCTTCACATTC | 108 | 98 |
| NR2B | NM_008171.3 | GAACAAGGAGAGGAAGTGGGAGAGG | CAGTCTCAGGACACATTCGAGGCC | 95 | 99 |
| TBP | NM_013684.3 | GATCAAACCCAGAATTGTTCTCC | GGGGTAGATGTTTTCAAATGCTTC | 106 | 97 |
| UBC | NM_019639.4 | CCACCAAGAAGGTCAAACAGG | CCCATCACACCCAAGAACAAG | 93 | 100 |

Primers for quantitative real time PCR analysis. The name of the amplified gene is indicated in the first column, followed by the accession number to the reference sequence. Primer sequences (forward and reverse) are shown in “forward primer” and “reverse primer” columns. In the last two columns the size (bp) of the amplification product and the primer efficiency (%) are indicated.
