# Supplementary material for: Decreased Hippocampal Neuroplasticity and Behavioral Impairment in an Animal Model of Inhalant Abuse
Source: Front Neurosci. 2018 Feb 6;12:35. doi: 10.3389/fnins.2018.00035 (PMC5810293; doi:10.3389/fnins.2018.00035)
Supplement: Supplementary file 2 [file Table2.docx]

# Supplementary Table 2

| Behavioral test | Treatment | Thinner concentration (ppm) | | | One-Way ANOVA | Bonferroni *post-hoc* | | |
| --- | --- | --- | --- | --- | --- | --- | --- | --- |
|  |  | Control | 300 | 600 |  | 300 vs Control | 600 vs Control | 300 vs 600 |
| TST | Acute | 105.8 ± 21.2 s | 105.0 ± 26.0 s | 99.0 ± 25.8 s | F_(2,27)_= 0.23 | t = 0.07 | t = 0.63 | t=0.55 |
|  | Subchronic | 93.7 ± 29.5 s | 127.5 ± 34.7 s | 125.4 ± 28.8 s | F_(2,27)_= 3.70* | t = 2.43 | t = 2.28 | t=0.15 |
|  | Chronic | 99.8 ± 28.2 s | 144.6 ± 30.4 s | 146.6 ± 33.9 s | F_(2,27)_= 7.33** | t = 3.24** | t = 3.38** | t=0.14 |
| FST  Climbing | Acute | 32.3 ± 7.5 s | 30.2 ± 6.9 s | 30.6 ± 6.8 s | F_(2,27)_= 0.24 | t = 0.66 | t = 0.52 | t=0.14 |
|  | Subchronic | 30.9 ± 5.8 s | 19.8 ± 5.5 s | 21.9 ± 6.9 s | F_(2,27)_= 9.43*** | t = 4.09** | t = 3.30** | t=0.79 |
|  | Chronic | 28.8 ± 7.4 s | 17.6 ± 7.5 s | 16.1 ± 6.9 s | F_(2,27)_= 9.07*** | t = 3.42** | t = 3.91** | t=0.49 |
| FST  Immobility | Acute | 54.0 ± 21.7 s | 47.9 ± 20.0 s | 48.5 ± 16.7 s | F_(2,27)_= 0.30 | t = 0.70 | t = 0.63 | t=0.07 |
|  | Subchronic | 48.0 ± 10.5 s | 48.3 ± 14.6 s | 58.0 ± 15.9 s | F_(2,27)_= 1.67 | t = 0.04 | t = 1.60 | t=1.56 |
|  | Chronic | 48.9 ± 11.2 s | 64.6 ± 13.0 s | 63.9 ± 14.3 s | F_(2,27)_= 4.71* | t = 2.71* | t = 2.60* | t=0.11 |
| OFT | Acute | 7.7 ± 2.6 % | 8.3 ± 3.2 % | 7.8 ± 2.9 % | F_(2,27)_= 0.09 | t = 0.41 | t = 0.07 | t=0.34 |
|  | Subchronic | 7.1 ± 2.0 % | 9.4 ± 2.7 % | 9.7 ± 2.3 % | F_(2,27)_= 3.66* | t = 2.15 | t = 2.49 | t=0.34 |
|  | Chronic | 7.0 ± 1.8 % | 13.8 ± 6.8 % | 13.3 ± 5.7 % | F_(2,27)_= 5.22* | t = 2.90* | t = 2.68* | t=0.22 |
| EPMT | Acute | 32.6 ± 10.9 % | 33.4 ± 11.5 % | 34.2 ± 12.1 % | F_(2,27)_= 0.06 | t = 0.15 | t = 0.30 | t=0.15 |
|  | Subchronic | 29.9 ± 7.1 % | 34.5 ± 8.1 % | 33.5 ± 5.0 % | F_(2,27)_= 1.26 | t = 1.51 | t = 1.17 | t=0.34 |
|  | Chronic | 30.4 ± 8.5 % | 53.5 ± 17.1 % | 53.3 ± 21.1 % | F_(2,27)_= 6.56** | t = 3.15* | t = 3.12* | t=0.03 |
| SPAT | Acute | 150.3 ± 48.3 s | 138.8 ± 50.6 s | 134.6 ± 52.6 s | F_(2,27)_= 0.26 | t = 0.51 | t = 0.70 | t=0.19 |
|  | Subchronic | 163.0 ± 24.7 s | 97.5 ± 60.7 s | 96.7 ± 59.3 s | F_(2,27)_= 5.57** | t = 2.87* | t = 2.91* | t=0.03 |
|  | Chronic | 145.8 ± 29.7 s | 77.4 ± 67.9 s | 51.7 ± 49.7 s | F_(2,27)_= 8.89** | t = 2.97* | t = 4.08** | t=1.11 |
| ORT | Acute | 68.2 ± 10.7 % | 63.7 ± 7.4 % | 67.1 ± 13.9 % | F_(2,27)_= 0.46 | t = 0.91 | t = 0.21 | t=0.71 |
|  | Subchronic | 61.9 ± 17.2 % | 58.7 ± 13.2 % | 41.2 ± 18.2 % | F_(2,27)_= 4.67* | t = 0.45 | t = 2.84* | t=2.40 |
|  | Chronic | 71.5 ± 10.0 % | 50.3 ± 7.5 % | 46.1 ± 16.9 % | F_(2,27)_= 12.62*** | t = 3.91** | t = 4.69*** | t=0.77 |

Statistical analysis of behavioral tests by one-way ANOVA followed by a Bonferroni *post-hoc* for multiple comparisons was used in each treatment (acute, subchronic and chronic). Abbreviations: TST: tail suspension test, FST: forced-swim test, OFT: open-field test, EPMT: elevated plus maze test, SPAT: step-through passive avoidance test, ORMT: object recognition memory test. The values reported are the mean ± SD (n=10 per group). ^*^P<0.05, ^**^P<0.01 and ^***^P<0.001 refers to the control (0 ppm) versus treated groups comparison.
